# Prevalence of Colonization With Antibiotic-Resistant Organisms in Hospitalized and Community Individuals in Bangladesh, a Phenotypic Analysis: Findings From the Antibiotic Resistance in Communities and Hospitals (ARCH) Study

**DOI:** 10.1093/cid/ciad254

**Published:** 2023-07-05

**Authors:** Fahmida Chowdhury, Syeda Mah-E-Muneer, Susan Bollinger, Aditya Sharma, Dilruba Ahmed, Kamal Hossain, Md Zakiul Hassan, Mahmudur Rahman, Daniel Vanderende, Debashis Sen, Palash Mozumder, Amin Ahmed Khan, Moushumi Sarker, Rachel M Smith, Ashley Styczynski, Ulzii-Orshikh Luvsansharav

**Affiliations:** International Centre for Diarrhoeal Disease and Research, Bangladesh (icddr, b), Dhaka, Bangladesh; International Centre for Diarrhoeal Disease and Research, Bangladesh (icddr, b), Dhaka, Bangladesh; Centers for Disease Control and Prevention (CDC), Atlanta, Georgia, USA; Centers for Disease Control and Prevention (CDC), Atlanta, Georgia, USA; International Centre for Diarrhoeal Disease and Research, Bangladesh (icddr, b), Dhaka, Bangladesh; International Centre for Diarrhoeal Disease and Research, Bangladesh (icddr, b), Dhaka, Bangladesh; International Centre for Diarrhoeal Disease and Research, Bangladesh (icddr, b), Dhaka, Bangladesh; International Centre for Diarrhoeal Disease and Research, Bangladesh (icddr, b), Dhaka, Bangladesh; Centers for Disease Control and Prevention (CDC), Atlanta, Georgia, USA; International Centre for Diarrhoeal Disease and Research, Bangladesh (icddr, b), Dhaka, Bangladesh; International Centre for Diarrhoeal Disease and Research, Bangladesh (icddr, b), Dhaka, Bangladesh; Mugda Medical College and Hospital, Dhaka, Bangladesh; Mugda Medical College and Hospital, Dhaka, Bangladesh; Centers for Disease Control and Prevention (CDC), Atlanta, Georgia, USA; Centers for Disease Control and Prevention (CDC), Atlanta, Georgia, USA; Centers for Disease Control and Prevention (CDC), Atlanta, Georgia, USA

**Keywords:** antibiotic resistance, prevalence, colonization, community, hospital

## Abstract

**Background:**

Low- and middle-income countries bear a disproportionate burden of antimicrobial resistance (AMR) but often lack adequate surveillance to inform mitigation efforts. Colonization can be a useful metric to understand AMR burden. We assessed the colonization prevalence of Enterobacterales with resistance to extended-spectrum cephalosporins, carbapenems, colistin, and methicillin-resistant *Staphylococcus aureus* among hospital and community dwellers.

**Methods:**

Between April and October 2019, we conducted a period prevalence study in Dhaka, Bangladesh. We collected stool and nasal specimens from adults in 3 hospitals and from community dwellers within the hospitals’ catchment area. Specimens were plated on selective agar plates. Isolates underwent identification and antibiotic susceptibility testing using Vitek 2. We performed descriptive analysis and determined population prevalence estimates accounting for clustering at the community level.

**Results:**

The majority of both community and hospital participants were colonized with Enterobacterales with resistance to extended-spectrum cephalosporins (78%; 95% confidence interval [95% CI], 73–83; and 82%; 95% CI, 79–85, respectively). Thirty-seven percent (95% CI, 34–41) of hospitalized patients were colonized with carbapenems compared with 9% (95% CI, 6–13) of community individuals. Colistin colonization prevalence was 11% (95% CI, 8–14) in the community versus 7% (95% CI, 6–10) in the hospital. Methicillin-resistant *Staphylococcus aureus* colonization was similar in both community and hospital participants (22%; 95% CI, 19–26 vs 21% (95% CI, 18–24).

**Conclusions:**

The high burden of AMR colonization observed among hospital and community participants may increase the risk for developing AMR infections and facilitating spread of AMR in both the community and hospital.

## Teaser

Colonization with extended-spectrum cephalosporin-resistant Enterobacterales was found among the majority of community and hospital participants in Dhaka, Bangladesh. Colonization prevalence with methicillin-resistant *Staphylococcus aureus* and colistin-resistant Enterobacterales was similar between community and hospital participants. Carbapenem-resistant Enterobacterales was detected more frequently among hospital participants, but a substantial number of community participants were also colonized with carbapenem-resistant Enterobacterales.

Antimicrobial resistance (AMR) has been established as the leading cause of infectious disease-related deaths globally with the greatest burden in low- and middle-income countries (LMICs) [1]. The spread of AMR in LMICs can be attributed to overstretched public health systems, insufficient access to diagnostics, overcrowding, inadequate access to safe water and sanitation, and lack of regulations for antibiotic use [2]. Among the most clinically important antibiotic-resistant pathogens are extended-spectrum beta-lactamase (ESBL)-producing Enterobacterales, carbapenem-resistant Enterobacterales (CRE), and methicillin-resistant *Staphylococcus aureus* (MRSA) [3–6]. The US Centers for Disease Control and Prevention and World Health Organization have declared these high-priority pathogens given the limited treatment options for infections caused by these organisms [7, 8]. Robust surveillance is essential for tracking the burden of these resistant pathogens and evaluating strategies to limit their spread.

AMR surveillance that relies on clinically diagnosed infections may not fully describe the true burden of AMR because these systems have the potential to be heavily affected by health-seeking behavior and availability and affordability of diagnostics [9]. Assessing the prevalence of resistant organisms through colonization screening can help inform surveillance efforts. Colonization with antibiotic-resistant bacteria often precedes infection, particularly among hospitalized patients who are at increased risk of developing infections resulting from invasive procedures and comorbidities [10, 11]. Although persons colonized with antibiotic-resistant organisms do not manifest any symptoms related to colonization, they are more likely to develop future resistant infections and can transmit resistant pathogens to other individuals [12]. Identifying rates of colonization with ESBL-producing Enterobacterales, CRE, colistin-resistant Enterobacterales (ColRE), and MRSA in hospitals and communities provides a useful opportunity to detect emergence and spread of antibiotic-resistant bacteria ahead of trends in clinical infections and provides a more holistic description of the burden of AMR in a geographic area [9, 13].

This study aimed to assess the prevalence of colonization with extended-spectrum cephalosporin-resistant Enterobacterales (ESCrE), CRE, ColRE, and MRSA among hospitalized and community-dwelling adult populations in Bangladesh.

## METHODS

### Study Design, Site, and Population

This community- and hospital-based period prevalence study was based in urban Dhaka city using previously described criteria [14]. We enrolled both community and hospital study participants from April to October 2019. We conducted the community enrollment in Kamalapur, a longitudinal community surveillance site of International Centre for Diarrhoeal Disease Research, Bangladesh. The hospital component was conducted in 3 hospitals (1 tertiary-level government hospital and 2 private hospitals) serving the same community population (Supplementary Figure 1). We enrolled patients from all inpatient departments except the pediatric and postoperative wards.

This study was part of the Antibiotic Resistance in Communities and Hospitals studies conducted across 6 countries to evaluate the population-based prevalence of colonization with clinically significant AMR organisms [14]. In addition to ESCrE, CRE, and MRSA prevalence detection as mentioned in the main Antibiotic Resistance in Communities and Hospitals protocol we incorporated ColRE to detect the prevalence in our study population in Bangladesh. No other deviation in methodology was considered from this published protocol.

### Community Sampling and Enrollment

We performed a 2-stage cluster sampling at the community level. At the first stage (cluster selection), 35 clusters of 666 clusters from 7 strata (differentiated based on geographical border) were selected based on probability proportional to population size sampling (Supplementary Figure 1). In the second stage (household selection), 30 households per cluster were selected by simple random sampling from the list of households obtained in the first stage. Finally, 1 adult was selected randomly among individuals in the household for participation. Eligibility criteria included individuals aged 18 years and older without fever, diarrhea, or cough at the time of the interview and specimen collection, and slept overnight in the household for at least 4 weeks before study enrollment. After obtaining written informed consent from the participants, we collected demographics and other relevant information.

### Hospital Sampling and Enrollment

The target sample size for enrollment at each hospital in the study was determined through probability proportional to patient population size sampling. Within hospitals, we continuously enrolled inpatients through simple random sampling from a list of hospitalized patients over a period of 7 months until we reached the target sample size [14]. Eligibility criteria included hospitalized patients aged 18 years or older without severe neutropenia or gastrointestinal bleeding. Field staff enrolled the hospitalized patients after obtaining written informed consent from the patient or, for unconscious or sedated patients, from an adult caretaker. Demographic and relevant health information was collected at the time of enrollment.

### Specimen Collection

Nasal swabs were collected from enrolled participants by study staff on the same day of enrollment using Copan Diagnostics Eswabs. After collection, nasal swabs were placed into liquid Amies transport media and kept at 4 °C until plating. Stool samples were self-collected in a sterile container or by an adult caretaker for unconscious patients. The day of stool sample collection typically occurred on the day after enrollment. After collection of the stool sample, study staff swabbed the stool with 3 swab sticks and placed swab sticks into 2 vials with Cary-Blair transport medium and 1 vial with buffered glycerol saline transport medium. Collected samples were kept at 2° to 8 °C and transported to the International Centre for Diarrhoeal Disease Research, Bangladesh, laboratory within 12 hours of collection.

### Laboratory Methods

Stool specimens were directly inoculated onto CHROMagar ESBL, mSuperCARBA, and CHROMagarCOL-*APSE* agar plates; nasal swabs were directly inoculated on CHROMagar MRSA agar plates. After 18–24 hours of incubation, up to 3 morphotypes per plate were selected to undergo bacterial identification and antibiotic susceptibility testing using bioMerieux Vitek 2 (gram-negative card: AST-N280; gram-positive card: AST-P628).

Isolates were classified as ESCrE when resistant to ceftriaxone and susceptible or intermediate to all tested carbapenems (imipenem, meropenem, ertapenem), CRE when resistant to at least 1 tested carbapenem, ColRE when resistant to colistin, or MRSA when resistant to oxacillin or positive by cefoxitin screen. We followed the Clinical and Laboratory Standards Institute (CLSI) 2019 breakpoints for antimicrobial susceptibility testing [15]. To characterize combined resistance patterns, we applied the difficult-to-treat resistance (DTR) definition to all *Escherichia coli, Klebsiella*, and *Enterobacter* isolates. DTR is defined as nonsusceptibility to all beta-lactams and fluoroquinolones against which the organism was tested [16].

### Data Analysis

Descriptive analyses were performed using STATA version 13. We estimated prevalence of ESCrE, CRE, ColRE, and MRSA colonization, applying 95% confidence intervals (CI) for hospitals and communities, adjusting for the 2-stage clustering effect using a nonparametric cluster bootstrap for the community. We used the statistical computing platform, R version 4.0.5, with the package “Complex-Upset” for presenting the antibiotic susceptibility results for the AMR phenotypes.

### Ethical Considerations

The study protocol (PR-18060) was reviewed and approved by the institutional review board of International Centre for Diarrhoeal Disease Research, Bangladesh (named the Research Review Committee and Ethical Review Committee).

## RESULTS

### Demographic Characteristics

We enrolled 768 community individuals and 743 hospitalized patients. All 768 community participants provided nasal swabs and 714 (93%) provided stool samples (Supplementary Figure 2). Of 743 enrolled hospital participants, all provided nasal swabs and 719 (97%) provided stool samples. Participants’ median age in years was 35 (interquartile range [IQR]: 25–40) among community participants and 40 (IQR: 30–55) among hospital participants. Two hundred and seventy-four (36%) community and 398 (54%) hospital participants were male.

### Clinical Characteristics and Hospitalization at Enrollment

Of the enrolled hospital participants, 54% (404) were from medicine wards, 15% (110) from surgery wards, 6% (43) from gynecology wards, 6% (43) from cardiology wards, 5% (39) from urology wards, and 14% (104) from other wards, including the intensive care unit (4, 1%). Among hospital participants, 44% (324) had 1 or more underlying chronic illnesses, including 42% (136) cardiovascular disease, 35% (112) diabetes, 22% (71) respiratory disease, 13% (41) kidney disease, 7% (24) liver disease, and 3% (10) other diseases. The median time between hospital admission and patient enrollment was 3 days (IQR: 2–6 days). However, 24% of the patients were enrolled within the first day of hospitalization, 18% on the second day, 14% on the third day, and 44% on the fourth day or later. Among participants with any of the antibiotic resistant phenotypes, the length of hospital stay before enrollment in the study was higher compared with participants without antibiotic-resistant phenotypes (3 days [IQR: 2–6 days] versus 2 days [IQR: 1–4 days]; *P* = .015).

### Prevalence of AMR Organisms

**Figure 1.**
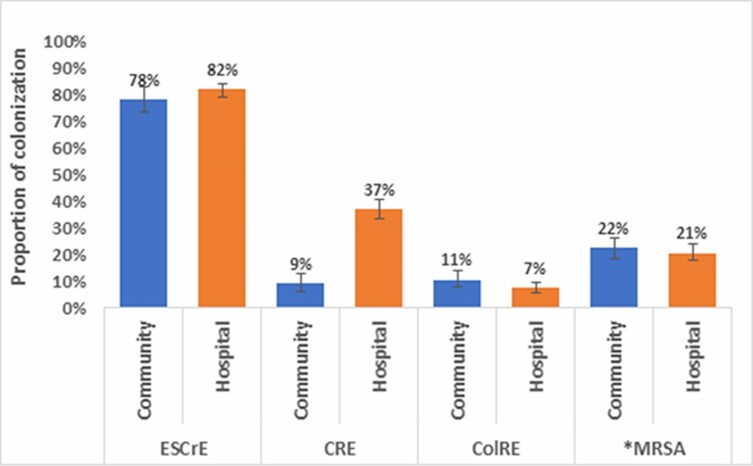
Prevalence of ESCrE,* CRE, ColRE, and MRSA colonization among hospital and community participants confirmed through VITEK 2, Bangladesh, 2019. ColRE, colistin-resistant Enterobacterales; CRE, carbapenem-resistant Enterobacterales; ESCrE, extended-spectrum cephalosporin-resistant Enterobacterales; MRSA, methicillin-resistant *Staphylococcus aureus*. *For MRSA, N = 768 in the community and N = 743 in the hospital. For ESCrE, CRE, and ColRE, N = 714 in the community and N = 719 in the hospital.

ESCrE colonization was high among both community and hospital participants (n = 558; 78% [95% CI, 73–83] vs n = 592; 82% [95% CI, 79–85], respectively) (Figure 1, Supplementary Table 1). However, CRE colonization prevalence was higher among the hospitalized patients compared with community individuals (n = 267; 37% [95% CI, 34–41] vs n = 66; 9% [95% CI, 6–13]), with nonoverlapping 95% CIs indicating a significant difference. ColRE colonization was similar between community and hospital participants (n = 76; 11% [95% CI, 8–14] vs n = 53; 7% [95% CI, 6–10]). MRSA colonization was also similar between the community and hospital participants (n = 172; 22% [95% CI, 19–26] vs n = 154; 21% [95% CI, 18–24], respectively). Only a small proportion of the community and hospital participants had no growth of these 4 specific AMR phenotypes (16% [111/714] vs 7% [49/719], respectively). Thirty percent (216/714) of community and 46% (328/719) of hospital participants were co-colonized with more than 1 of the target AMR organisms (Supplementary Table 2).

### Pathogen-Specific Antibiotic-Resistant Pattern

**Figure 2.**
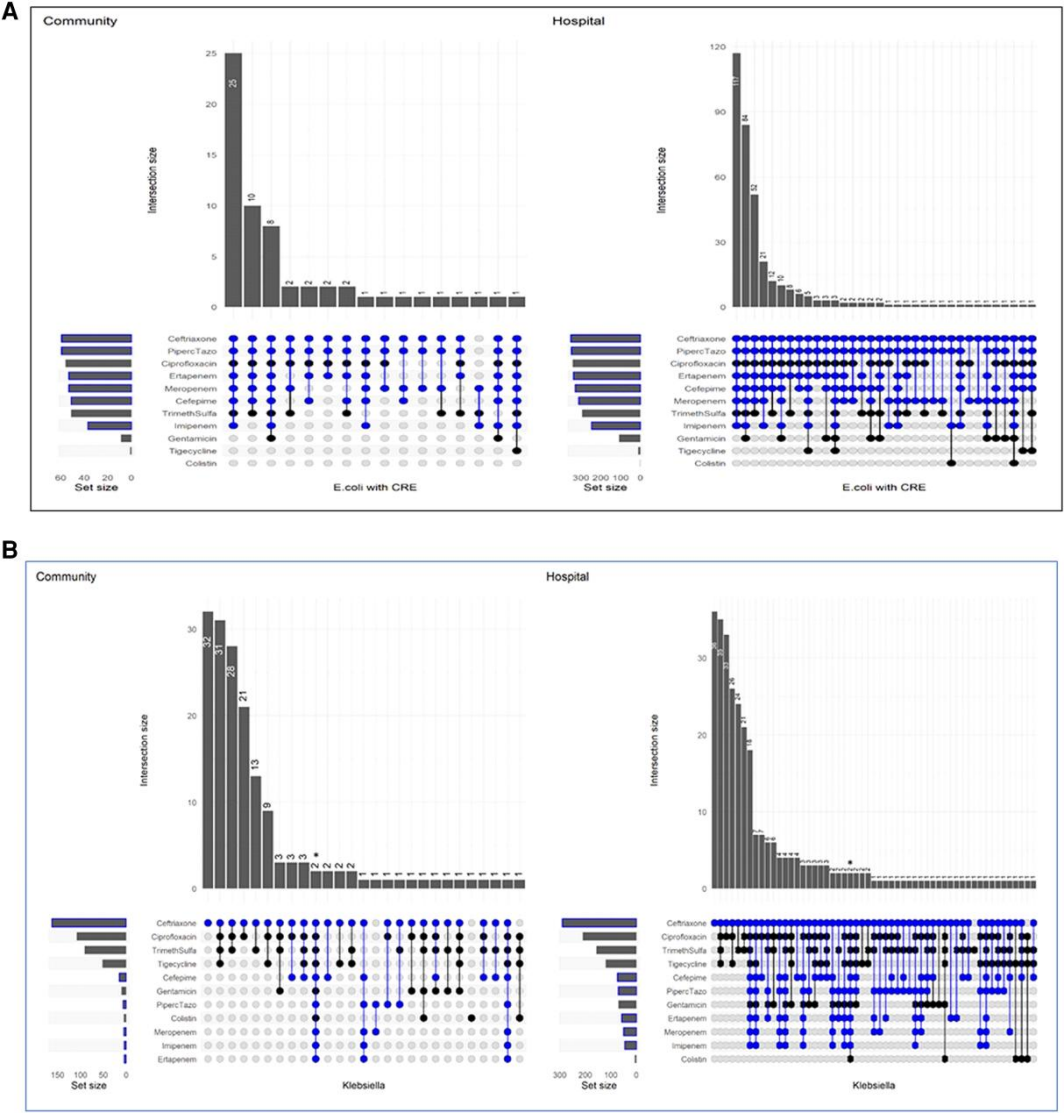
*A*, Antibiotic resistance pattern among *Escherichia coli* (*E. coli*) with CRE phenotype in communities (N = 60) and hospitals (N = 351). The top portion of each graph shows the frequency of different antibiotic resistance patterns, which are depicted beneath each of the columns. The horizontal bars show the frequency of resistance against a given antibiotic. Blue color dots indicate B-lactam antibiotics (ceftriaxone, cefepime, piperacillin-tazobactam, imipenem, meropenem, and ertapenem). *B*, Antibiotic resistance among *Klebsiella* spp. in communities (N = 169) and hospitals (N = 293). The top portion of each graph shows the frequency of different antibiotic resistance patterns, which are depicted beneath each of the columns. The horizontal bars show the frequency of resistance against a given antibiotic. Blue color dots indicate B-lactam antibiotics (ceftriaxone, cefepime, piperacillin-tazobactam, imipenem, meropenem, and ertapenem). *Pan-resistant isolate.

**Figure 3.**
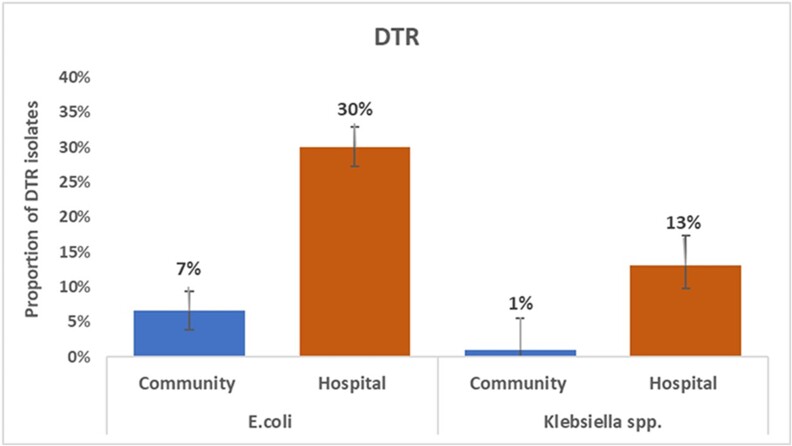
Prevalence of difficult-to-treat resistance (DTR) phenotypes in *Escherichia coli* and *Klebsiella* spp. isolates from community and hospital participants, Bangladesh, 2019. For *E. coli,* N = 731 in the community and N = 1028 in the hospital. For *Klebsiella* spp., N = 186 in the community and N = 305 in the hospital.

Among 2442 Enterobacterales isolates recovered, *E. coli* was the most prevalent (75% [n = 1759]; 731 in communities and 1028 in hospitals) organism followed by *Klebsiella* spp. (20% [n = 491]; 186 in communities and 305 in hospitals). Resistance to multiple antibiotic classes was more common among hospital isolates compared with the community except among *E. coli* with CRE and ESCrE phenotypes (Figure 2*A* and Supplementary Figure 3*A*). Two isolates of *Klebsiella pneumoniae* identified from hospital participants and 2 isolates of *K. pneumoniae* from community participants demonstrated resistance to all antibiotic classes against which they were tested (Figure 2*B* ). Among all *E. coli* isolates, the DTR phenotype was found in 30% (95% CI, 27.5–33.1) of isolates from hospital participants and 7% (95% CI, 3.9–9.3) of isolates from community participants (Figure 3). Similarly, among *Klebsiella* spp. isolates, the DTR phenotype was found in 13% (95% CI, 9.8–17.4) of isolates from hospital participants and 1% (95% CI, 0–4.6) of isolates from community participants. No *Enterobacter* isolates demonstrated DTR phenotypes. For MRSA isolates, similar resistance patterns were observed among community and hospital participants (Supplementary Figure 3*B*).

## DISCUSSION

Our study revealed a high prevalence of AMR colonization among both hospital and community participants in a densely urban setting in Bangladesh. Only CRE colonization was significantly higher among hospital participants compared with the community. Frequent colonization with ESCrE, ColRE, and MRSA among community participants demonstrates the importance of communities as reservoirs for AMR in Bangladesh. However, multidrug resistance was higher in the hospital setting, as evidenced by the greater abundance of colonization with organisms bearing the DTR phenotype among hospitalized participants.

The high prevalence of ESCrE colonization we detected in the community (78%) was similar to findings previously reported in Bangladesh that showed 82% of healthy infants and 68% of adults in rural households were colonized with *E. coli* resistant to third-generation cephalosporins [17, 18]. However, ESCrE colonization prevalence in Bangladesh, as demonstrated in this study, remains much higher than global and Southeast Asia–specific estimates of ESBL intestinal colonization in both community (17.6% and 35.1%, respectively) and healthcare settings (21.1% and 32.9%, respectively) [19]. There are several reasons why Bangladesh may have higher rates of intestinal carriage with ESCrE organisms. Extensive use of third-generation cephalosporins [20, 21] along with widespread usage of penicillins or other cephalosporins may also propagate ESCrE if it is already widely circulating. The abundant availability and use of these antibiotics in the absence of any enforced prescription requirements may be contributing to the genesis and transmission of ESCrE in communities and hospitals. In addition to antibiotic use in humans, studies have reported up to 100% usage of antibiotics in poultry, both for treatment and prophylactic purposes and have detected AMR pathogens including ESBL-producing *E. coli* in commercial chicken, fish, livestock, and animal origin food in Bangladesh [22]. Consumption of this food, contact with domestic animals, or work in aquaculture/agriculture may also be important factors behind the high prevalence of ESCrE pathogens among community individuals. Additionally, less than one-half the population in Bangladesh has access to safely managed sanitation services, which can result in environmental contamination with resistant gram-negative pathogens [23]. Moreover, community AMR colonization can result from exposure to healthcare facilities during outpatient visits or while visiting hospitalized family members, which warrants further exploration in future studies [24, 25]. It is crucial to identify the major potential drivers of antibiotic resistance in the community to plan and implement directed prevention and mitigation efforts against ESCrE outside the hospital to successfully mitigate the impact of AMR across sectors.

Although CRE colonization was much less frequent than ESCrE colonization, its circulation at any level is concerning given the limited number of treatment options against pathogens harboring this type of resistance. Previously reported rates of community CRE colonization globally have found the highest rates in the Asia-Pacific Region [26]. CRE was significantly more common among hospitalized patients in our study. Although this could be a result of patient-level factors, it also indicates likely nosocomial transmission and the existence of selective pressures (eg, antibiotic usage) within healthcare settings that may amplify the spread of carbapenem resistance. Hospitals in Bangladesh have been found to have inadequate infection prevention and control (IPC) practices [25] and thus may contribute to the spread of drug-resistant organisms. Therefore, it is crucial to strengthen IPC practices alongside implementation of antimicrobial stewardship programs to combat AMR.

The problem of multidrug resistance is further highlighted by the frequent recovery of isolates with a DTR phenotype. Although this study only assessed colonization and not infection with DTR organisms, the large percentage of organisms with a DTR phenotype suggests that this phenotype is likely present in clinical infections in the hospitals. Moreover, infections caused by these organisms have been shown to be associated with worse clinical outcomes and impose the use of less effective and more toxic antibiotics, if available [27]. Further data on the magnitude of DTR infections and outcomes in LMICs such as Bangladesh are critical.

We found that ColRE colonization was higher within community individuals compared with hospitalized individuals [28, 29]. Colistin resistance is concerning because it is the last-line antibiotic in many LMICs, including Bangladesh, where newer antibiotics are unavailable or ineffective because of different resistance mechanisms [30]. The spread of ColRE is not likely to be attributable to the healthcare setting because it is infrequently used in Bangladesh clinical contexts [31]. However, colistin is commonly used in Bangladeshi chicken production cycles, and the effects can be clearly seen by the abundance of ColRE among feed animals and chicken droppings [32]. This may explain the higher frequency of resistance in the community. This demonstrates why the World Health Organization recommends reserving last-line antibiotics, such as colistin, for treatment of highly resistant infections through coordinated approaches such as regulatory oversight of antibiotic use and access in both human and animal sectors [33].

For the past 2 decades, MRSA has transitioned from a primarily healthcare-associated pathogen to one that has become prevalent in the community globally [34]. In both the community and hospital participants of our study, MRSA colonization was similar. However, it is higher than the rates typically seen in high-resource settings as well as other LMICs [35–37]. Although the reasons for high rates of MRSA colonization in Bangladesh are unclear, similar factors contributing to the spread of gram-negative pathogens may be facilitating spread of MRSA colonization in hospitals and communities. Although there are still more treatment options for infections caused by MRSA compared with multidrug-resistant gram-negative infections, ongoing monitoring of trends in MRSA prevalence is important for minimizing its health impacts.

Our study was limited to examining the prevalence of resistance only among colonizing isolates, and it is unknown how differences in colonization predict differences in the incidence of infection. Another limitation was the relatively short time points into hospitalization when patients were screened, which could have led to lower estimates of colonization. A third limitation of our study was the method used to determine colistin susceptibility. Determination of accurate colistin MIC is challenging, particularly in LMICs where recommended methods (eg, broth microdilution) are resource intensive and often not available. The Vitek platform, used in this study, is known to produce both major and, in particular, major errors when compared with reference broth microdilution, resulting in an undercalling of resistance and hence is not recommended by CLSI [28, 29]. Given this, true ColRE prevalence in our study sites may be higher than reported here. Additionally, we enrolled participants from a single, urban geographic region, so our findings may not be generalizable across a broader population of Bangladesh.

Our study is the first population-based estimate of AMR colonization in Bangladesh. The high prevalence of AMR colonization among community and hospital participants identified in our study is cause for concern, along with findings from other studies showing rising rates of AMR in clinical isolates in Bangladesh [38]. Better understanding the risk factors for colonization with multidrug-resistant organisms may provide an upstream opportunity to intervene before they lead to infection and further spread, incurring heavy societal and economic costs. Prevention activities based only in healthcare facilities will likely not be sufficient to address the full scope of the growing AMR crisis, given the widespread community colonization. Comprehensive strategies involving diverse stakeholders from healthcare providers to policy makers are urgently needed. These data can be used to support and advocate for the establishment of robust surveillance platforms using a One Health approach and the development of stronger IPC programs and antimicrobial stewardship programs to reduce the burden of AMR.

## Supplementary Data

Supplementary materials are available at *Clinical Infectious Diseases* online. Consisting of data provided by the authors to benefit the reader, the posted materials are not copyedited and are the sole responsibility of the authors, so questions or comments should be addressed to the corresponding author.

## Supplementary Material

ciad254_Supplementary_Data

## Notes

***Supplement sponsorship.*** This article appears as part of the supplement “The Evolving Challenges of Antibiotic Resistance in Low- and Middle-Income Countries: Priorities and Solutions,” sponsored by the U.S. Centers for Disease Control and Prevention, and Health Security Partners.
