# Supplementary material for: Prevalence of Colonization With Antibiotic-Resistant Organisms in Hospitalized and Community Individuals in Bangladesh, a Phenotypic Analysis: Findings From the Antibiotic Resistance in Communities and Hospitals (ARCH) Study
Source: Clin Infect Dis. 2023 Jul 5;77(Suppl 1):S118–24. doi: 10.1093/cid/ciad254 (PMC10321696; doi:10.1093/cid/ciad254)
Supplement: ciad254_Supplementary_Data [file ciad254_supplementary_data.zip › Supplementary figures and tables_05.04.23_clean.docx]

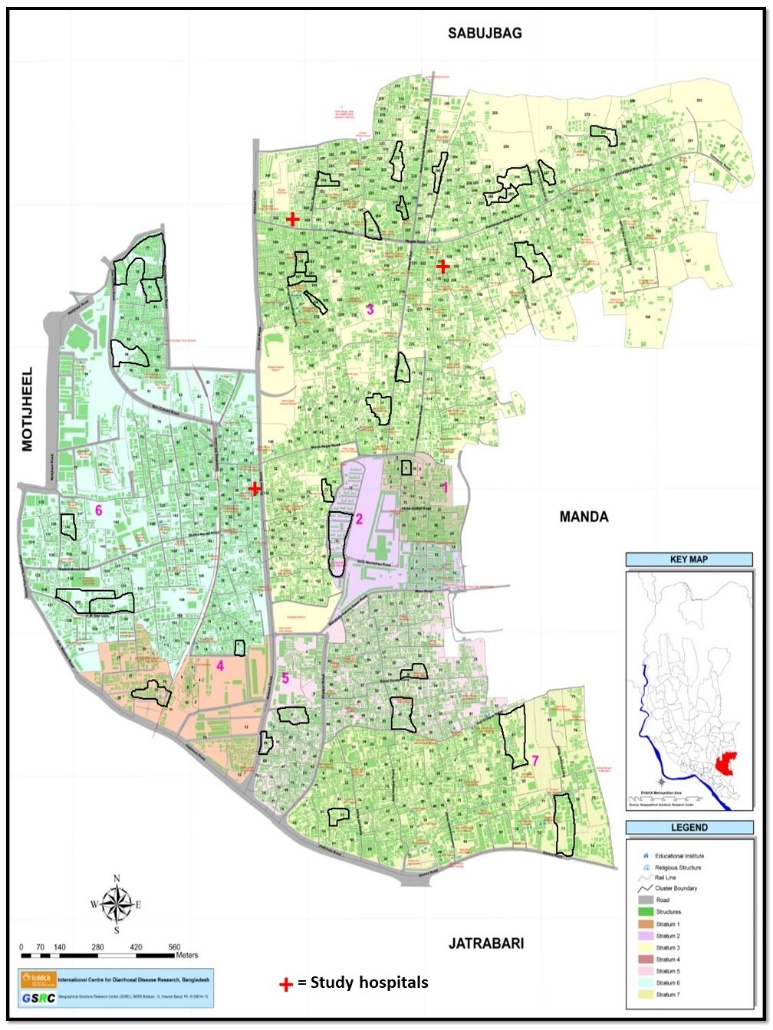


Note: Areas with black borders are randomly selected community clusters from which study participants were recruited.

**Supplementary Figure 1:** Study sites in Dhaka city with selected clusters and hospitals


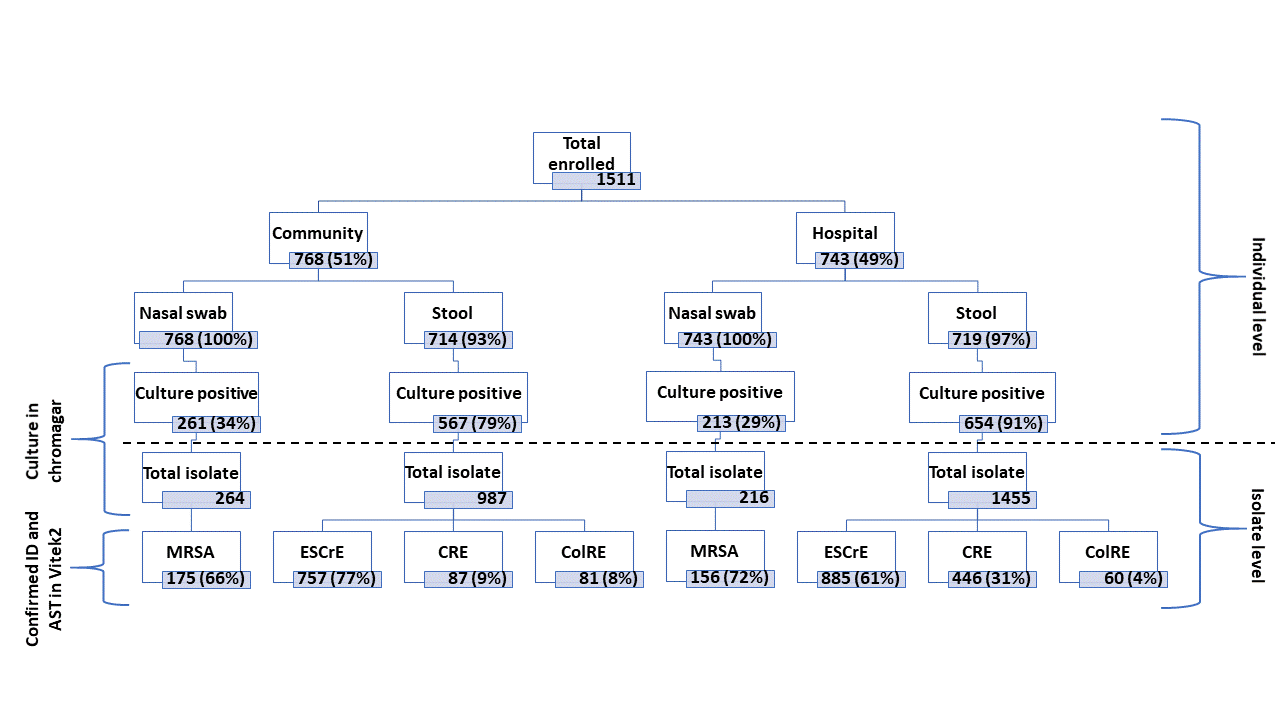


Note: Extended-spectrum cephalosporin-resistant Enterobacterales (ESCrE), carbapenem-resistant Enterobacterales (CRE), colistin-resistant Enterobacterales (ColRE), and methicillin-resistant *Staphylococcus aureus*

**Supplementary Figure 2: Culture positivity rates by specimen type and isolate-level phenotypes using VITEK®2, Bangladesh, 2019**

**Supplementary Table 1:** **Prevalence of** **ESCrE, CRE, ColRE and MRSA colonization among hospital and community participants confirmed through VITEK**® **2, Bangladesh, 2019**

|  | **Confirmed identification of antibiotic-resistant isolates in VITEK**® **2** | | | |
| --- | --- | --- | --- | --- |
|  | Community (N=714) | | Hospital (N=719) | |
| **Phenotypic resistance** | n (%) | 95% CI | n (%) | 95% CI |
| ESCrE | 558 (78.1) | (73.3-83.0) | 592 (82.3) | (79.2-84.8) |
| CRE | 66 (9.2) | (6.1-12.7) | 267 (37.1) | (33.5-40.6) |
| ColRE | 76 (10.6) | (7.9-14.0) | 53 (7.4) | (5.7-9.5) |
| MRSA* | 172 (22.4) | (18.6-26.4) | 154 (20.7) | (18.0-23.8) |

Note: ESCrE (Extended-spectrum cephalosporin-resistant *Enterobacterales*), CRE (carbapenem-resistant *Enterobacterales*), ColRE (colistin-resistant *Enterobacterales*) and MRSA (methicillin-resistant *Staphylococcus aureus*)

* For MRSA, N=768 in Community and N=743 in Hospital

**Supplementary Table 2: Co-colonization of ESCrE, CRE, ColRE, and MRSA among hospital and community participants confirmed through VITEK 2, Bangladesh, 2019**

| **Phenotypic resistance** | **Community**  **(N=714)**  **n (%)** | **95%CI** | **Hospital**  **(N=719)**  **n (%)** | **95%CI** |
| --- | --- | --- | --- | --- |
| ESCrE+CRE | 58 (8.1) | (5.2-11.3) | 203 (28.2) | (25-32) |
| ESCrE+ColRE | 70 (9.8) | (7.1-13.0) | 51 (7.1) | (5.4-9.2) |
| ESCrE+MRSA | 119 (16.7) | (13.0-20.5) | 122 (17.1) | (14.4-19.9) |
| CRE+ColRE | 17 (2.4) | (1.0-4.0) | 25 (3.5) | (2.3-5.1) |
| CRE+MRSA | 16 (2.2) | (1.0-3.7) | 50 (7.0) | (5.3-9.1) |
| ColRE+MRSA | 15 (2.1) | (0.8-3.5) | 9 (1.3) | (0.6-2.4) |
| ESCrE+CRE+ColRE | 16 (2.2) | (0.9-3.8) | 23 (3.2) | (2.1-4.8) |
| ESCrE+CRE+MRSA | 11 (1.5) | (0.5-2.8) | 37 (5.1) | (3.7-7.0) |
| ESCrE+ColRE+MRSA | 13 (1.8) | (0.6-3.2) | 8 (1.1) | (0.6-2.2) |
| ESCrE+CRE+ColRE+MRSA | 2 (0.3) | (0-0.9) | 6 (0.8) | (0.4-1.8) |

Note: ESCrE (Extended-spectrum cephalosporin-resistant Enterobacterales), CRE (carbapenem-resistant Enterobacterales), ColRE (colistin-resistant Enterobacterales), and MRSA (methicillin-resistant *Staphylococcus aureus*)

**Supplementary Figure 3 A: Antibiotic –resistance among *Escherichia coli*** ***(E. coli)* with ESCrE phenotypes in communities (N=579) and hospitals (N=637)**


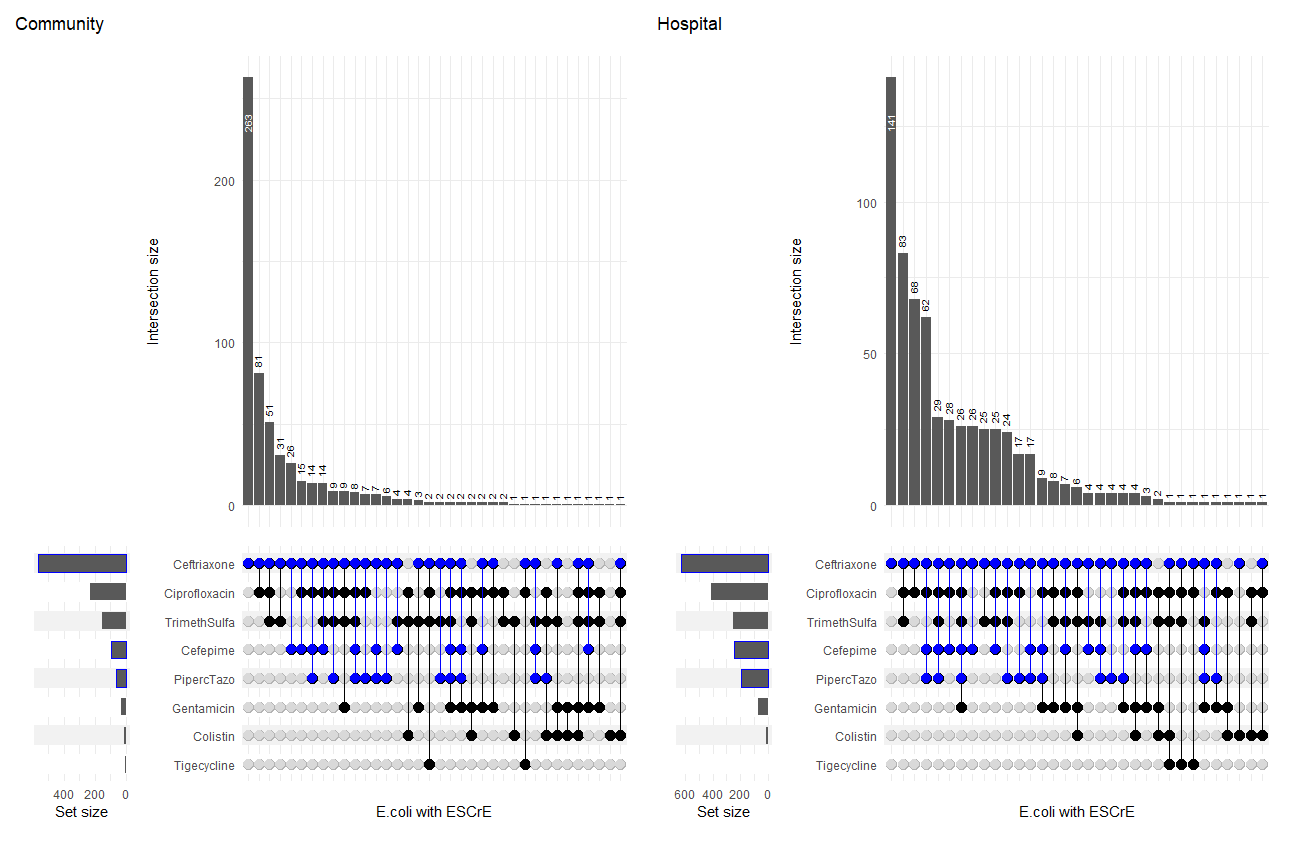


The top portion of each graph shows the frequency of different antibiotic resistance patterns, which are depicted beneath each of the columns. The horizontal bars show the frequency of resistance against a given antibiotic.

Blue color dots indicate B-lactam antibiotics (ceftriaxone, cefepime, piperacillin-tazobactam, imipenem, meropenem, and ertapenem).

**Supplementary** **Figure 3 B: Antibiotic resistance among MRSA isolates in communities (N=175) and hospitals (N=156), Bangladesh, 2019**


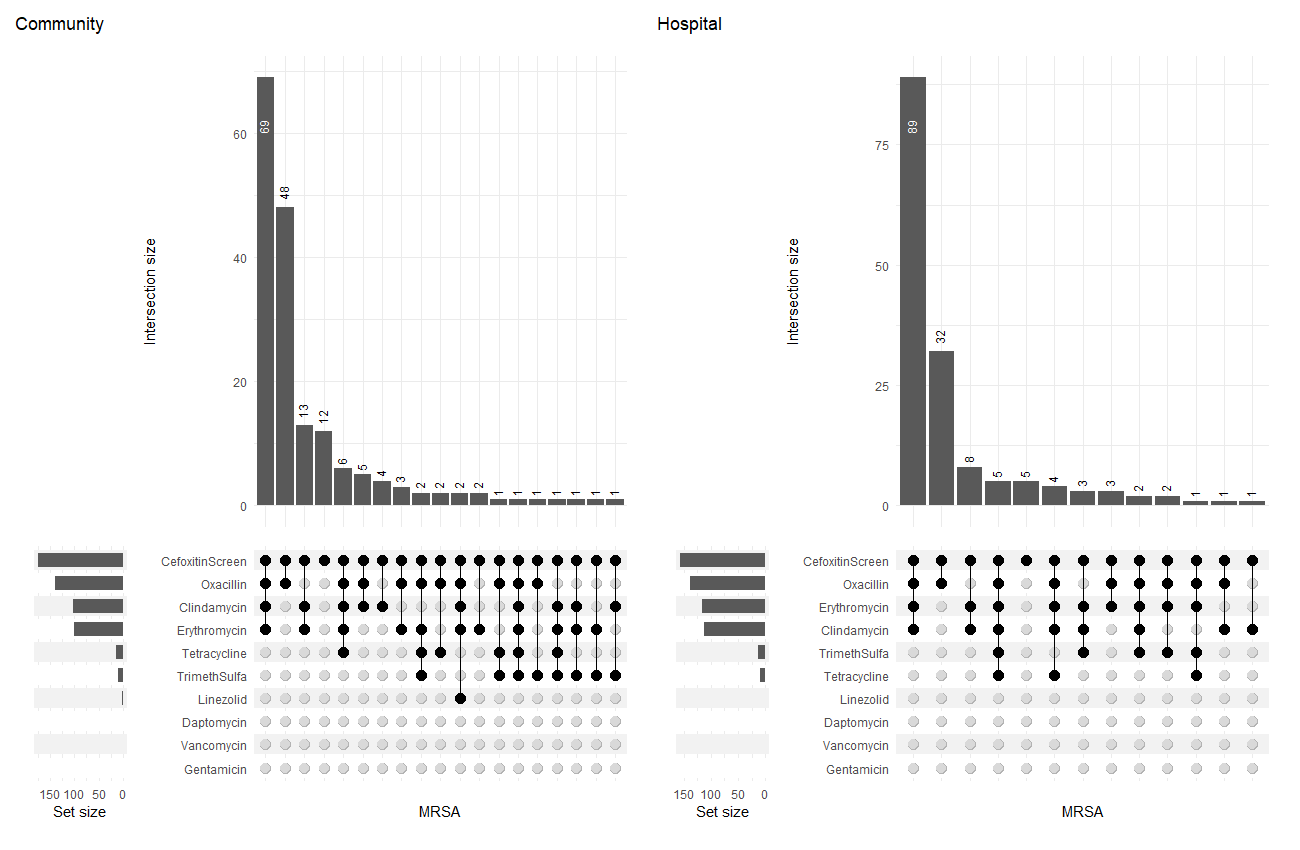


The top portion of each graph shows the frequency of different antibiotic resistance patterns, which are depicted beneath each of the columns. The horizontal bars show the frequency of resistance against a given antibiotic.

Blue color dots indicate B-lactam antibiotics (ceftriaxone, cefepime, piperacillin-tazobactam, imipenem, meropenem, and ertapenem).
