# Supplementary figures and images for: Prevalence of Colonization With Antibiotic-Resistant Organisms in Hospitalized and Community Individuals in Bangladesh, a Phenotypic Analysis: Findings From the Antibiotic Resistance in Communities and Hospitals (ARCH) Study
Source: Clin Infect Dis. 2023 Jul 5;77(Suppl 1):S118–24. doi: 10.1093/cid/ciad254 (PMC10321696; doi:10.1093/cid/ciad254)

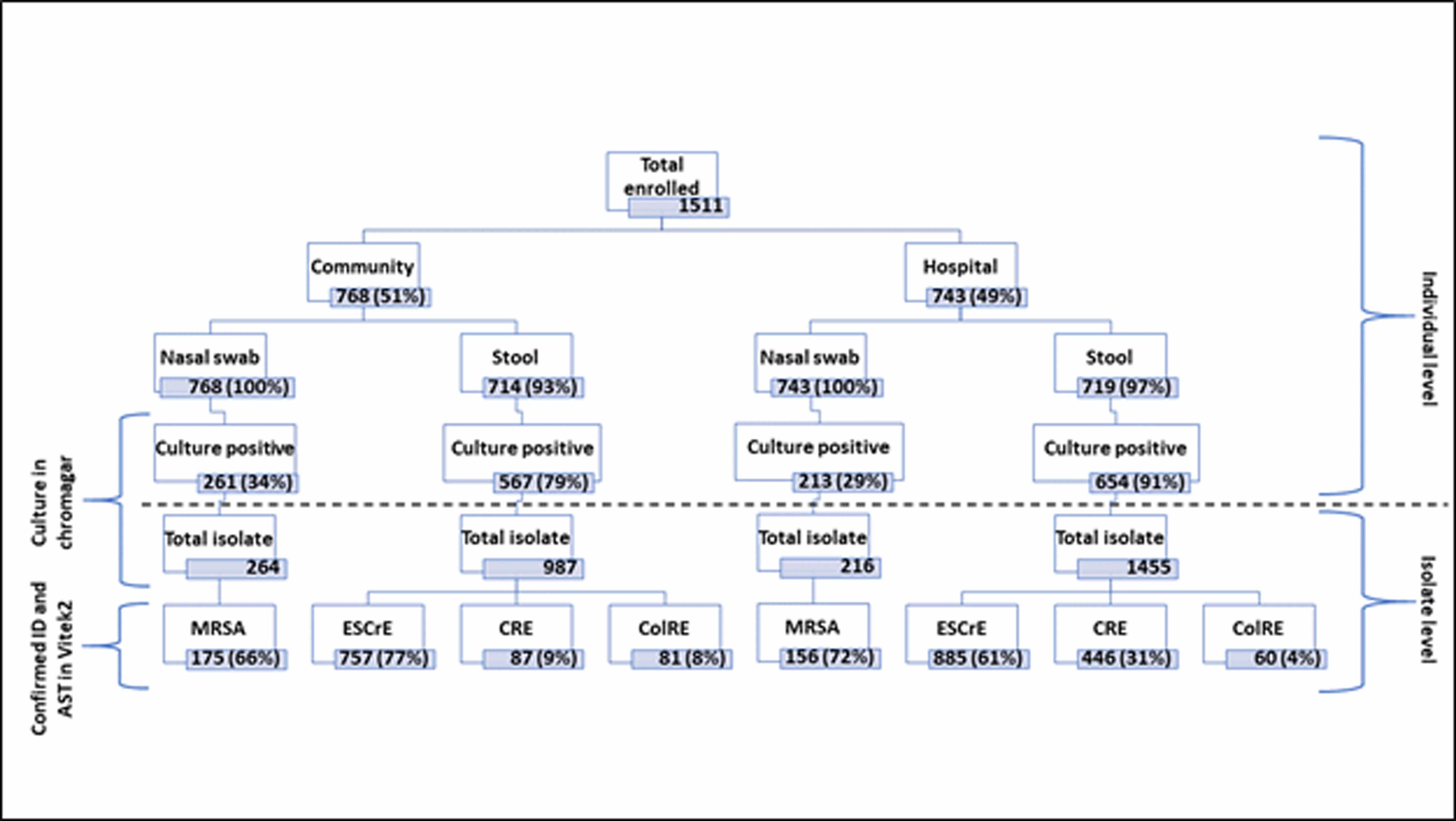

Supplement: ciad254_Supplementary_Data [file ciad254_supplementary_data.zip › Supplementary Figure 2.jpg]

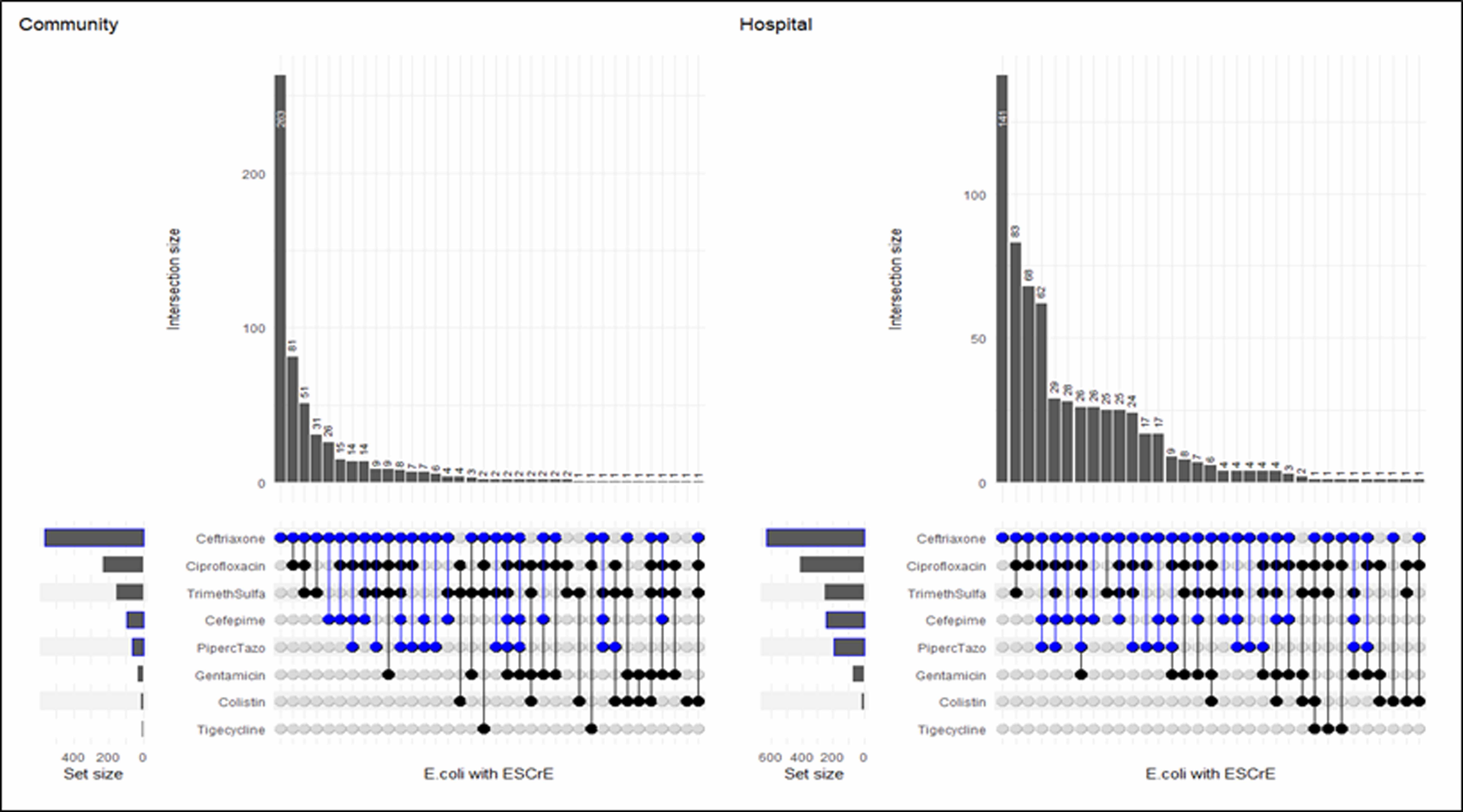

Supplement: ciad254_Supplementary_Data [file ciad254_supplementary_data.zip › Supplementary Figure 3 A.jpg]

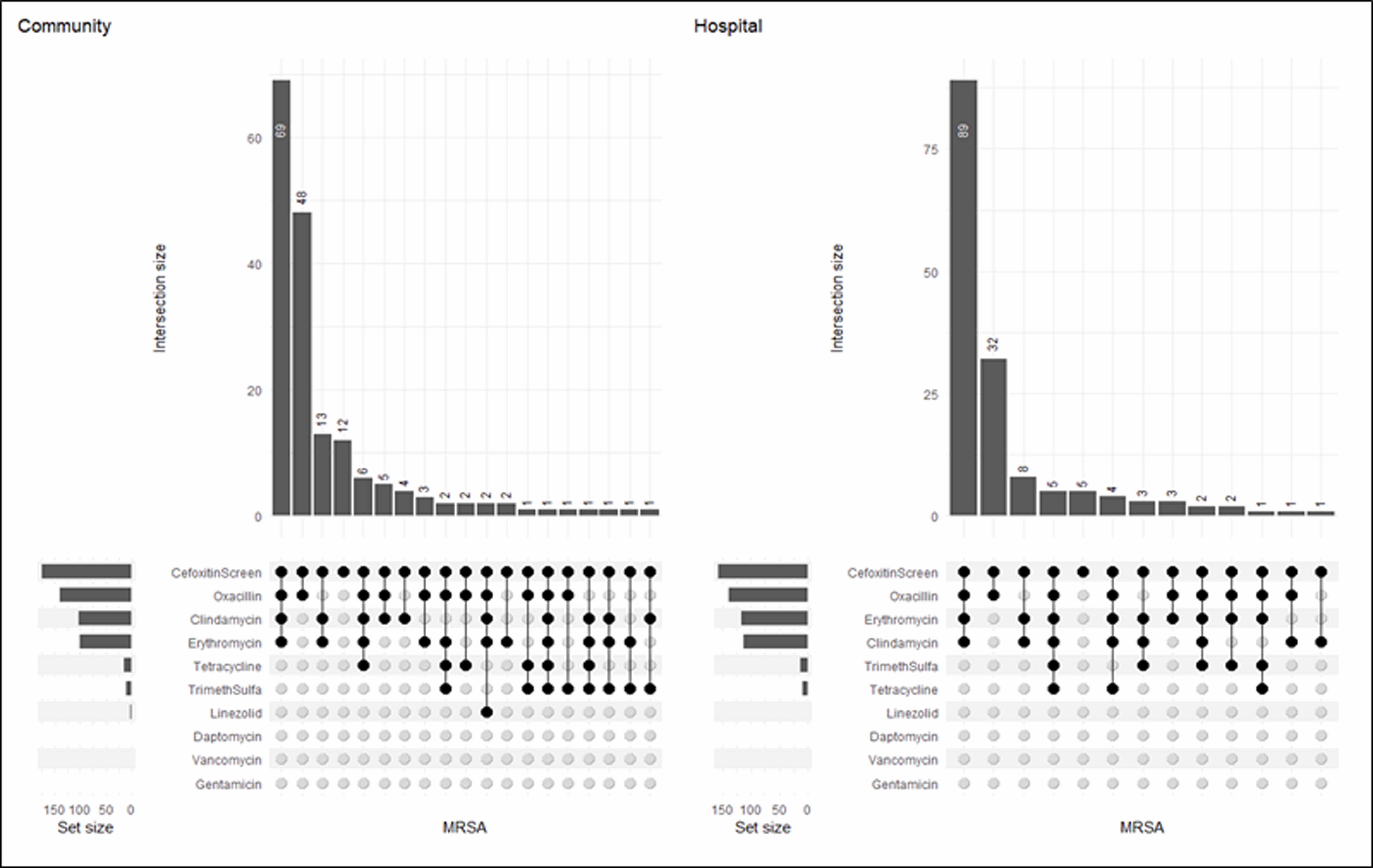

Supplement: ciad254_Supplementary_Data [file ciad254_supplementary_data.zip › Supplementary Figure 3 B.jpg]
